# Embryological Divergence and Molecular Mechanisms in Thoracic and Abdominal Aortic Aneurysms: Bridging Developmental Biology and Clinical Insights

**DOI:** 10.3390/biom15121654

**Published:** 2025-11-26

**Authors:** Mathias Van Hemelrijck, Petar Risteski, Laura Rings, Milan Milojevic, Héctor Rodríguez Cetina Biefer, Omer Dzemali

**Affiliations:** 1 Department of Cardiac Surgery, Municipal Hospital Triemli, 8055 Zurich, Switzerland; mathias.vanhemelrijck@usz.ch (M.V.H.); petar.risteski@usz.ch (P.R.); laura.rings@usz.ch (L.R.); hector.rodriguez@usz.ch (H.R.C.B.); 2 Department of Cardiac Surgery, University Hospital Zurich, 8091 Zurich, Switzerland; milan.milojevic@usz.ch; 3 Department of Cardiovascular Research, Dedinje Cardiovascular Institute, 11000 Belgrade, Serbia; 4 Center for Translational and Experimental Cardiology (CTEC), Department of Cardiology, University Hospital Zurich, 8952 Zurich, Switzerland

**Keywords:** aorta, aortic aneurysm, thoracic aortic disease, abdominal aortic disease, genetic variants, aortopathy, clinical management, patient-centred cardiovascular medicine

## Abstract

Aortic aneurysms are complex, predominantly asymptomatic vascular diseases with distinct incidence patterns depending on anatomical localisation. The incidence of thoracic aortic aneurysms (TAAs) has moderately increased, whereas that of abdominal aortic aneurysms has declined, primarily due to public health measures. Undiagnosed or poorly managed aneurysms are at significant risk of progression to acute aortic syndrome, with high associated mortality. The embryological origins of the aorta may have a substantial impact on its structural, cellular, and functional heterogeneity. Specifically, smooth-muscle cells (SMCs) in the thoracic aorta are derived from cardiac neural crest and mesodermal cells, whereas abdominal aortic SMCs originate from the paraxial and splanchnic mesoderm. To explore these developmental and regional distinctions, we conducted a narrative review based on targeted literature retrieval and expert curation, highlighting how these distinctions might potentially influence susceptibility to aneurysms and their clinical presentation. Histological differences, such as the number of lamellar units and the presence or absence of vasa vasorum, could further explain regional vulnerability. Molecular mechanisms underlying aneurysm formation include inflammation, oxidative stress, extracellular matrix degradation, phenotypic switching, and dysregulated signalling pathways, notably transforming growth factor-beta (TGF-β) and angiotensin II. Genetic mutations significantly contribute to TAAs, with genes involved in the elastin–contractile unit and TGF-β signalling pathways playing pivotal roles. However, the complex interplay between genetic susceptibility and risk factors explains why some patients develop aneurysms while others do not. Clinical management strategies have evolved, emphasising early risk stratification, surveillance, and timely surgical intervention, guided increasingly by genetic profiling and segment-specific molecular understanding. Advances in genomic technologies, biomarker identification, and computational modelling promise to enhance individualised care. Bridging developmental biology, molecular genetics, and clinical practice is crucial for improving outcomes in patients with aortic aneurysms, thereby reinforcing a multidisciplinary approach to patient-centred cardiovascular medicine.

## 1. Introduction

Aortic aneurysms are predominantly asymptomatic, showing variable incidence and prevalence that depend significantly on their anatomical localisation, with global epidemiological data indicating an overall increasing trend [1,2]. The incidence rate of thoracic aortic aneurysms (TAAs) has moderately increased to up to 10 per 100,000 person-years, while abdominal aortic aneurysms (AAAs) have notably declined in recent years, with prevalence rates decreasing from a range of 4% to 5% to approximately 1.5% to 3% [3,4,5,6]. The annual incidence of AAAs is 2.5–6.5 per 1000 person-years [7]. This decrease is primarily attributed to improved public health initiatives, particularly smoking cessation, cardiovascular risk factor management, as well as widespread ultrasound screening in the older adult population [8], with elevated systolic blood pressure emerging as the current leading modifiable risk factor for aneurysm formation [1]. Genetic susceptibility to aneurysm formation explains why patients with diagnosed aortic ectasia or aneurysm are more likely to have a dilatation at any other aortic segment [9]. While cardiovascular disease and atherosclerosis-related comorbidities play notable roles in AAA development, their influence on TAAs remains comparatively limited. The complex interplay between genetic susceptibility (caused by identified mutations in aneurysm-related genes) and environmental or risk factors is key, explaining the varying clinical features seen in affected families with specific genetic defects.

Undiagnosed or inadequately managed aortic aneurysms are associated with a significant risk of progression to acute aortic syndrome (AAS). The clinical spectrum of AAS includes acute aortic dissection, intramural haematoma, penetrating aortic ulcers, and aortic rupture, each associated with high mortality rates of up to 75% depending on the study population and treatment experience [10]. Prevalence estimates for AAS vary widely, ranging from 0.2% to 0.8% in autopsy studies [11] to incidence rates of up to 17.6 per 100,000 person-years in clinical registries [12]. In the United States, the CDC ranks aortic aneurysms, dissections, and ruptures as the 19th leading cause of death, accounting for approximately 43,000–47,000 deaths annually [13]. Moreover, population-based projections forecast a 42% increase in aortic disease-related mortality by 2030 [8], emphasising the need for proactive risk identification and preventative strategies. Recent findings have confirmed that aneurysm diameter correlates directly with an exponential increase in AAS, reinforcing the importance of early detection and timely medical or surgical intervention to mitigate the risk of catastrophic outcomes [14].

Elective surgical repair significantly reduces both short- and long-term mortality risks compared to emergency presentation, irrespective of localisation [8]. Historically, the surgical criteria for treating TAAs were extrapolated from AAA data, overlooking numerous distinct morphological and clinical considerations including connective tissue disorders, consequences of aortic valve disease, and hereditary thoracic aortic aneurysms. Recent advances in molecular-, genetic-, and population-level research have supported the application of refined criteria, including diameter thresholds and aortic wall integrity, to guide earlier surgical intervention in selected patients [14]. Still, debate persists over whether these thresholds should be lowered further to improve outcomes in high-risk groups, especially given the significantly improved clinical outcomes of elective procedures in experienced centres [15]. Optimal management therefore relies on early risk stratification, longitudinal surveillance, and appropriate intervention timing to avoid emergent presentations and reduce associated morbidity [8,16]. 

Furthermore, the recent recognition of the aorta as a complex, functionally distinct organ has reinforced the importance of accounting for segment-specific embryological origins and divergent pathological mechanisms when selecting treatments [8,17]. Each segment, derived from different embryonic lineages, such as the neural crest and mesoderm, exhibits unique cellular architecture, biomechanical properties, and disease susceptibility, which might help explain the biological difference between thoracic and abdominal aortic aneurysms. Moreover, there is growing interest in elucidating the molecular and genetic drivers of aneurysm formation, particularly connective tissue disorders such as Marfan, Ehlers–Danlos, and Loeys–Dietz syndromes [18]. These advances are driving a shift toward patient-centred, evidence-based medicine, utilising genetic profiling and individualised surveillance strategies to achieve pre-emptive interventions.

Given these considerations, a comprehensive understanding of aortic aneurysm pathogenesis requires a multidisciplinary perspective, integrating embryology, vascular biology, histopathology, clinical genetics, and therapeutic planning to support optimal patient care. This narrative review synthesises current knowledge across these domains to present a unified mechanistic concept of the “aortic organ,” emphasising how structural and developmental heterogeneity could drive segment-specific susceptibility to aneurysm formation and inform diagnostic, prognostic, and therapeutic strategies.

## 2. Embryological Development and Structural Differences

### 2.1. Embryological Development and Organogenesis

The development of the aortic organ and cardiovascular system is a complex and tightly regulated embryological process involving multiple distinct cell populations. The heart tube originates from both the first heart field, which forms most of the left ventricle, and the second heart field in the pharyngeal mesoderm, which contributes myocardium to the right ventricle and both the proximal outflow tract and intrapericardial great arteries. After looping, a single outflow tract connects the ventricle to an aortic sac. Second-heart-field cells and migrating cardiac neural crest populate the outflow tract and aortic sac, where endocardial cushions form and remodel. Septation of the distal outflow tract and the aortic sac produces the intrapericardial arterial trunks, the ascending aorta and the pulmonary trunk, while the remainder of the proximal outflow tract becomes the subaortic and subpulmonary outlets and the semilunar valves. The aortic sac is then remodelled into right and left horns that integrate with the pharyngeal arch arteries to generate the proximal arch vessels and the definitive intrapericardial ascending aorta; caudal to the fourth thoracic level, the paired dorsal aortae fuse to form the descending aorta. Region-specific lineage contributions underlie the later heterogeneity of the aortic wall: smooth-muscle cells (SMCs) of the ascending aorta and arch are largely derived from the neural crest with additional second-heart-field input, whereas abdominal aortic smooth-muscle cells originate predominantly from the paraxial/splanchnic mesoderm; endothelial cells arise from the splanchnic mesodermal endothelium, and adventitial fibroblasts and perivascular cells are derived from local mesenchyme with a neural-crest contribution in the thoracic segment. This proximodistal sequence and lineage map replace the previous “truncus arteriosus/bulbus cordis” framework and align the developmental narrative with contemporary usage and mechanisms relevant to aortopathy.

Cardiac neural crest cells (CNCCs) originate from the dorsal neural tube via epithelial–mesenchymal transition, and then proliferate and migrate into pharyngeal arches II, III, and VI and the distal outflow tract/aortic sac. CNCCs are pivotal to the formation of the aortopulmonary septum, the separation of the intrapericardial arterial trunks, and elements of the semilunar valves [19]. They also modulate smooth-muscle differentiation within the tunica media. Consequently, smooth-muscle lineage is region-specific: the aortic root is largely second-heart-field-derived [20], and the descending thoracic and abdominal aortae arise mainly from the paraxial and splanchnic mesoderm, respectively [21,22], while descending thoracic and abdominal aorta SMCs originate from paraxial mesoderm-derived somite and the splanchnic mesoderm, respectively [21].

Although direct evidence is currently lacking, developmental lineage differences may contribute to regional variations in aortic wall architecture and behaviour and consequently be used to explain the abdominal predilection for atherosclerosis and the thoracic localisation of many HTADs, as discussed in the following sections.

### 2.2. Aortic Wall: Structural and Histological Differences

Histologically, the aortic wall consists of three primary layers: the tunica intima, the tunica media, and the tunica adventitia. The intima, the innermost layer, directly interfaces with blood flow and is composed of endothelial cells, a supporting basement membrane, and an internal elastic lamina. It functions as a selective barrier, maintaining vascular homeostasis and regulating haemodynamics. The tunica adventitia, the historically underappreciated outermost layer, was previously viewed as merely a fibroblastic tissue embedded within an extracellular matrix (ECM). However, recent insights have identified its complex composition, including fibroblasts, adipocytes, mast cells, macrophages, lymphocytes (T- and B-cells), dendritic cells, collagen- and elastin-rich ECM, vasa vasorum, nerves, lymphatics, and progenitor cells [23]. Beyond providing structural support and nutrient supply, the adventitia plays a critical role in modulating inflammatory responses, facilitating repair mechanisms following injury, and regulating vascular remodelling [23]. The tunica media, situated between the intima and adventitia, forms the thickest aortic layer, comprising SMCs surrounded by a mix of elastin, collagen fibres, proteoglycans, and adhesive proteins. The media layer provides the aorta’s structural integrity and elasticity, accommodating dynamic arterial pressures [24]. Structurally, the aorta is the largest elastic artery, characterised by lamellar units, fundamental functional elements consisting of SMC surrounded by elastic fibres and collagen [25]. Within these units, SMCs are uniquely arranged in a “herringbone” pattern, optimising their viscoelastic properties and evenly distributing biomechanical stress [13].

**Figure 1:**
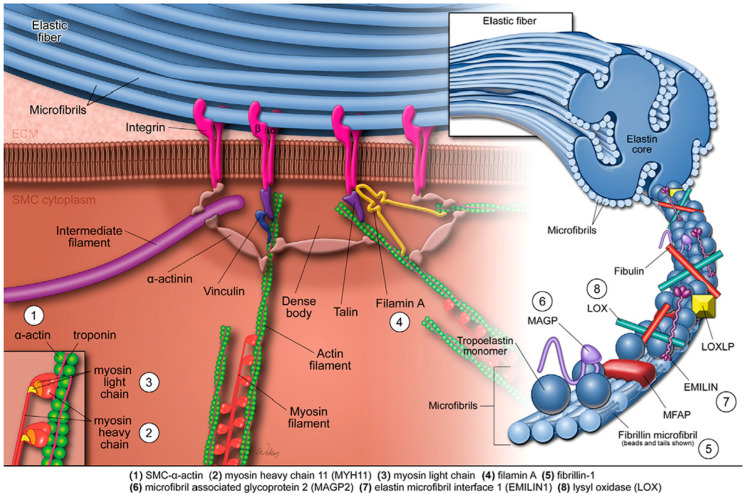
The elastin–contractile unit and mutations affecting aortic contractile function. The elastin–contractile unit is a specialised configuration composed of elastic fibres, focal adhesions (or dense plaques) in the plasma membrane, and contractile filaments within SMCs. Elastic fibres consist of a central elastin core surrounded by microfibrils composed of fibrillin, MAGPs, EMILIN1, fibulins, and other glycoproteins. The SMC contractile apparatus includes actin-containing thin filaments, myosin-containing thick filaments, and associated regulatory proteins. Mechanical stimuli are transmitted from the elastic fibre through membrane-bound focal adhesions, then via anchoring or actin-linking proteins to the contractile apparatus, ultimately triggering SMC contraction. Genetic thoracic aortic aneurysms and dissections are associated with mutations in genes encoding structural and regulatory components of the elastin–contractile unit. These include smooth-muscle α-actin (ACTA2), myosin heavy chain 11 (MYH11), myosin light chain kinase (MYLK), filamin A (FLNA), fibrillin-1 (FBN1), MAGP2, EMILIN1, and lysyl oxidase (LOX). EMILIN1: elastin microfibril interfacer protein 1; MAGPs: microfibril-associated glycoproteins; SMCs: smooth-muscle cells. Reproduced from Shen and LeMaire [23] with permission from Elsevier.

Elastic fibres, synthesised and secreted by SMCs, form an intricate network comprising an elastin core encased by microfibrils, such as elastin microfibril interfacer protein 1 (EMILIN-1), fibrillin, microfibril-associated glycoproteins (MAGPs), fibulins, and other glycoproteins. Together with focal adhesions, dense plaques, and contractile filaments inside the SMCs, elastic fibres create an elastin-contractile unit that is essential for mechanotransduction. This unit communicates biomechanical stimuli from elastic fibres directly to the SMCs’ contractile apparatus, which includes actin-rich thin filaments, myosin-rich thick filaments, and associated regulatory proteins (Figure 1) [26]. Collagen and elastin comprise approximately 60% of the aorta’s dry weight, with higher elastin-to-collagen ratios observed in thoracic segments compared to an inverse ratio in abdominal segments, underscoring the greater elasticity of the thoracic aorta compared to its stiffer abdominal counterpart [25].

Comparative mammalian histology indicates that circumferential wall tension per medial lamellar unit is broadly species-invariant and that the proportional distribution of medial thickness, lamellar units, and vasa vasorum is conserved [27]. In the thoracic elastic aorta, the inner avascular zone of the media is nourished by diffusion from the lumen, whereas the vasa vasorum supply the outer media; by contrast, the human abdominal aorta lacks medial vasa vasorum and contains fewer lamellar units for its calibre than the thoracic aorta and than the abdominal aortas of other mammals [27,28]. Thoracic segments typically possess ≈55–60 lamellar units, whereas abdominal segments average ≈25–30 [27,28]. The growth of the human abdominal aorta predominantly occurs through the widening of existing lamellae rather than the creation of new units [29]. These architectural features likely increase stress per lamellar unit and may predispose the abdominal segment to aneurysm formation; progressive dilatation further disrupts the media and can compromise nutrient delivery, promoting pathological remodelling [27,28,29]. 

These within-species histological differences may reflect adaptive evolutionary changes that enable the aorta to function under increased mechanical stress.

## 3. Molecular and Cellular Mechanisms of Aneurysm Formation

Aortic aneurysms can be broadly categorised into sporadic and genetically derived forms. Sporadic aneurysms, accounting for approximately 80% of cases, involve complex mechanisms that are not yet fully understood, while genetically derived aneurysms have been linked to specific genetic mutations. The formation and progression of aneurysms is a chronic process characterised by inflammation, mechanical stress, transcriptional regulation, and oxidative stress. These factors interact to degrade the tunica media, dilating and weakening the aortic wall [30]. This structural remodelling, primarily driven by a loss of SMCs, ECM degradation, and fragmentation of elastic fibres, compromises the aorta’s structural integrity and elasticity [28]. Specifically, the breakdown of elastic fibres disrupts the elastin–contractile unit, diminishing the flexibility of collagen fibres and increasing arterial stiffness. Elastin degradation also activates the transforming growth factor-beta (TGF-β) pathway. SMCs play critical roles beyond mere contraction; they actively maintain ECM homeostasis [31]. Hypothetically, this developmental heterogeneity might influence the distinct susceptibility of aortic segments to aneurysm formation, with segment-specific pathophysiological mechanisms involving inflammation, matrix metalloproteinase (MMP) imbalance, immune modulation, and altered TGF-β signalling [28]. Currently, there is no evidence to support the altered expression of aneurysm-related genes across the different segments of the healthy aorta. Inflammatory activation in the aortic wall may be initiated and amplified by smoking, arterial hypertension and haemodynamic overload, atherosclerosis, and genetic predisposition [32].

### 3.1. Endothelial Cell Dysfunction

As mentioned previously, endothelial cells (ECs) play major roles, not only as barriers and in maintaining vascular homeostasis, but also in regulating SMCs. In abnormal pathological conditions, they may even demonstrate destructive properties in the aorta. Studies in mice have shown that deficiency of the angiotensin 1 receptor reduces the formation of angiotensin-II-induced aneurysms [33], and that EC-specific ROS production increases susceptibility to aortic dissection [34]. 

Specific EC dysfunction occurs in abdominal and thoracic aneurysms. In AAAs, where turbulent flow, shear stress, and endothelial injury are present, ECs switch from their protective antithrombotic role to a procoagulant role, undertaking processes such as binding and activating platelets and leukocytes, and secreting von Willebrand factor [35]. This procoagulant status may lead to intraluminal thrombus formation, which is predominantly present in AAAs, and impaired permeability of the endothelial wall, permitting low-density proteins to penetrate the intimal space. In TAAs, EC function is dysregulated, promoting SMC phenotypic switching [36]. A recent study showed that EC tight junctions may be a potential target for preventing aneurysm formation [37].

### 3.2. Smooth-Muscle-Cell Apoptosis and Phenotypic Switching

SMC loss significantly contributes to aneurysm development through apoptosis, which is triggered under cyclic mechanical stress conditions. These occur when the endoplasmic reticulum (ER) stress response is activated via the transcription factor C/EBP homologous protein (CHOP) [31]. Multiple factors, including aldosterone, mast cell-derived chymase and tryptase, TGF-β, protein kinase C, and p53, contribute to this apoptotic process [24]. Conversely, elevated high-density lipoprotein (HDL) can exert a protective effect by mitigating angiotensin II (AT-II)-induced apoptosis.

Under stress or inflammatory conditions, SMCs undergo phenotypic switching, transitioning from a contractile to a synthetic, inflammatory, or osteogenic phenotype, which allows for adaptation but also contributes to dysfunction [38]. Phenotypic switching typically results in the expression patterns of SMC genes and ECM proteins becoming altered, accompanied by increased expression of pro-inflammatory molecules such as MMPs; however, the exact triggers remain unclear. In TAAs, SMCs switch to synthetic and migratory phenotypes, whereas in AAAs they become inflammatory [39,40]. It has also been shown that these SMCs can dedifferentiate into MSC-like cells, and from this state further differentiate into osteoblasts. Consequently, bone-like proteins, mineralisation, calcification and ossification of the aortic wall are more likely to occur in AAAs [41].

### 3.3. Pro-Inflammatory Cells

Inflammatory cells significantly influence aneurysm pathogenesis. Macrophages produce pro-inflammatory cytokines (e.g., IL-6, TNF-α) and MMPs, with an imbalance between the tissue-destructive M1 and resolution-promoting M2 subtypes frequently observed in aneurysms [24]; cytokine-mediated monocyte recruitment is also prominent in AAAs [42]. Neutrophils contribute to ECM degradation via elastase and MMPs, while mast cells in the AAA adventitia exacerbate tissue damage by promoting apoptosis and MMP activation through the secretion of chymase and tryptase [43]. Myofibroblasts derived from endothelial-to-mesenchymal transition contribute to repair, but may also propagate inflammation through increased MMP activity. T-lymphocytes, particularly CD4+ subsets, are also implicated in aneurysmal inflammation. Pro-inflammatory T-helper 1, 2, and 17 cells promote inflammatory cascades involving cytokines (IL, IFN-γ, TNF-α), macrophages, and MMPs, thereby exacerbating aneurysm formation. Conversely, regulatory T-cells may mitigate inflammation, suggesting a protective role [44].

### 3.4. MMPs and Tissue Inhibitors of MMPs

Matrix metalloproteinases, produced by endothelial cells and categorised as elastases, collagenases, or gelatinases, critically regulate ECM dynamics [45]. Specifically, MMP-2 and MMP-9 are prominent in aneurysms; MMP-9 is elevated in both TAAs and AAAs, as well as in Marfan syndrome, whereas MMP-2 is predominantly increased in AAAs [46,47]. Interestingly, MMP-17 appears protective, with loss-of-function pathogenic variants associated with inherited thoracic aneurysms [48].

Tissue inhibitors of MMPs (TIMPS) are key endogenous inhibitors of MMP activity and are influenced by factors that stimulate medial degeneration. SMCs primarily produce TIMP-1 and TIMP-2 in the aortic walls; TIMP-1 broadly inhibits MMPs, particularly MMP-9, while TIMP-2 has a dual role, capable of both inhibiting and activating MMP-2 and MMP-9, depending on the context. The balance between MMPs and TIMPs regulates ECM turnover, and disruptions in their ratio, reflected in the so-called proteolytic index, have been implicated in both TAA and AAA pathogenesis [46].

### 3.5. AT-II Pathway

Angiotensin II infusion has been shown to promote aortopathies in mice models [49]. Angiotensin II is the most potent aneurysmal factor and acts through two receptors, angiotensin receptor 1 (AT1R) and angiotensin receptor 2 (AT2R), with opposing effects. Activation of AT1R promotes vascular pathology by stimulating Rho kinases, TNF-α, ERK1/2, JNK, and p38, as well as the production of reactive oxygen species (ROS), partly through increased activity of NADPH oxidases (NOXs) [50]. This increase in ROS drives SMC phenotypic switching, inflammation, fibroblast proliferation, and adverse aortic remodelling. In an animal (mice) model, Das et al. demonstrated that oxidative stress acts differently depending on anatomical location due to locoregional antioxidant differences in signalling pathways [51]. Furthermore, when pharmacological inhibition was provided, aneurysm formation was blocked, suggesting a potential pharmacological target.

Mitochondria also contribute to oxidative stress through ROS generation, amplifying the downstream effects of AT1R signalling [52]. Contrarily, angiotensin II receptor blockers (ARBs), inhibit this pathway by suppressing AT1R-mediated responses, including pressure-induced myofibroblast activation and vascular remodelling [53]. 

### 3.6. TGF-β Pathway

**Figure 2:**
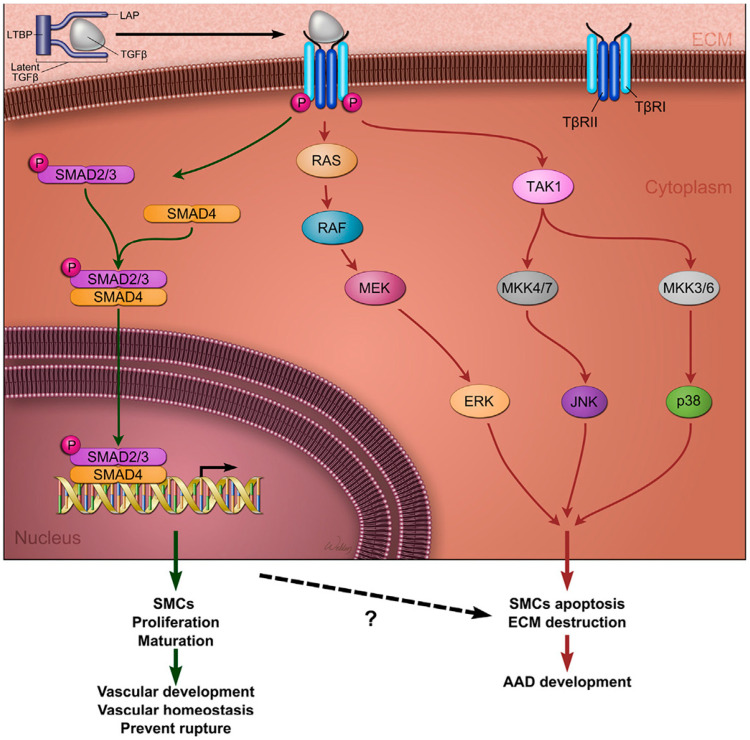
Transforming growth factor-β signalling in aortic aneurysms and dissections. TGF-β ligands are produced as part of a large latent complex composed of mature TGF-β, a LAP, and an LTBP. TGF-β signals through both canonical and non-canonical pathways. In the canonical pathway, TGF-β binds to its type II receptor (TβRII), which recruits and phosphorylates the type I receptor (TβRI). Activated TβRI then phosphorylates receptor-regulated SMADs (R-SMADs), primarily SMAD2 and SMAD3. These SMADs translocate to the nucleus, where they bind to the promoters of target genes and regulate transcription. Canonical TGF-β signalling supports normal aortic development and maintains vascular homeostasis. Mutations in genes encoding TGF-β ligands, receptors, or SMAD proteins can cause thoracic aortic aneurysms and dissections. In non-canonical pathways, the activated TGF-β receptor complex initiates signalling cascades via mediators such as TGF-β-activated kinase 1 (TAK1), p38 mitogen-activated protein kinase (p38 MAPK), extracellular signal-regulated kinase (ERK), JUN N-terminal kinase (JNK), and nuclear factor-κB (NF-κB). These non-canonical pathways contribute to aortic wall degradation and the pathogenesis of AAD. LAP: latency-associated peptide; LTBP: latent TGF-β-binding protein; TGF-β: Transforming growth factor-β. Reproduced from Shen and LeMaire [23] with permission from Elsevier.

TGF-β is a multifunctional cytokine that plays a central role in cardiovascular morphogenesis and the regulation of ECM homeostasis [54]. It is secreted as part of a large latent complex composed of mature TGF-β, latency-associated peptide (LAP), and latent TGF-β-binding proteins (LTBPs). TGF-β signalling occurs through both canonical and non-canonical pathways, influencing SMC differentiation, ECM synthesis, and remodelling. In the canonical pathway, TGF-β binds to its receptors (TGFBR1 and TGFBR2), leading to the phosphorylation and activation of SMAD2/3 and SMAD4. These SMAD proteins then translocate to the nucleus, where they regulate the expression of target genes associated with ECM maintenance and SMC stabilisation. This pathway is generally considered to be protective in aortic development. Conversely, the non-canonical pathway activates downstream mediators such as ERK, JNK, and p38 MAP kinases, which promote apoptosis, inflammation, and pathological remodelling [24,55]. When canonical signalling is deficient or dysregulated, compensatory TGF-β overexpression may excessively activate the non-canonical pathway, contributing to ECM degradation, SMC phenotypic switching, and the formation of aortic aneurysms (Figure 2) [56,57].

In addition to TGF-β, several other signalling pathways contribute to aortic wall remodelling and aneurysm progression. These include (i) β-arrestin-2, which upregulates MMP-2 and MMP-9; (ii) endothelin-1, associated with increased ROS generation, MMP-2 expression, and inflammatory cell infiltration; (iii) aldosterone–mineralocorticoid receptor signalling, which promotes salt-induced elastin degradation, SMC apoptosis, and inflammation; (iv) kinin B2-receptor, implicated in ATII-induced SMC phenotypic switching and MMP activation; (v) apelin, with emerging roles in vascular homeostasis; and (vi) low-density lipoprotein receptor-related protein 1 (LRP1), which plays a protective role in maintaining SMC function and vessel integrity [24].

### 3.7. Atherosclerosis-Driven Inflammation

The presence of atherosclerosis in AAAs, and to a lesser degree in TAAs, is evident; cardiovascular risk factors, such as arterial hypertension, smoking, high LDL levels, and low HDL levels, are common in these patients [28,58,59,60,61]. While atherosclerosis develops in parallel with or secondary to aneurysmal dilatation [62], several mice models have shown that a high-fat diet can induce hypercholesterinaemia and consequently increase the incidence of AT-II-induced AAAs [63]. 

### 3.8. MicroRNAs (miRNAs)

Several miRNAs are known to cause TAAs, AAAs, or dissections [24]. Through degradation and translational repression, target mRNAs are post-transcriptionally silenced. Multiple miRNAs have been identified in mouse models: silencing miR-205/miR-712 prevented AAA development by decreasing angiotensin-II-induced MMP activity and inflammation [64]; blocking miR-29b in Marfan mice prevented wall apoptosis, ECM deficiencies, and aneurysm formation in their ascending aortas [65]. Recently, miR15-a has been linked to AAAs, with increased tissue and plasma/serum levels correlating with more severe phenotypes [66].

### 3.9. Genetic Predisposition

Approximately 20% of patients with thoracic aortic aneurysm or dissection have a family history suggesting hereditary predisposition [13,24,67,68]; within this familial subset, pathogenic variants in known aneurysm-related genes can be identified in about 20% of cases. Both thoracic and abdominal aortic aneurysms have substantial genetic components, with significant overlap in susceptibility genes [69].

Recent studies indicate this substantial genetic contribution to AAAs, with twin studies suggesting a heritability of up to 70% [70]. It has been demonstrated that relatives of patients with AAAs show a higher incidence of TAAs, suggesting genetic susceptibilities that are not yet fully understood [71]. A recent meta-genome-wide association study (GWAS) identified 141 susceptible loci related to AAAs [72], a substantial increase in disease loci compared to previous studies. Furthermore, GWAS analysis suggested a positive correlation between AAAs and 20 cardiometabolic disorders, such as those linked to lipid metabolism [73], showing that aortic aneurysms share common traits with cardiovascular diseases such as arterial hypertension, hypercholesterinaemia, or inflammation. These findings also suggest potential targets for pharmacological interventions. Furthermore, GWAS findings can refine risk prediction models and polygenic risk scores that estimate individual genetic susceptibility to aneurysms, and can also serve as targets for novel therapies for AAA aimed at limiting aneurysm growth and reducing the risk of cardiovascular events [74]. 

**Figure 3:**
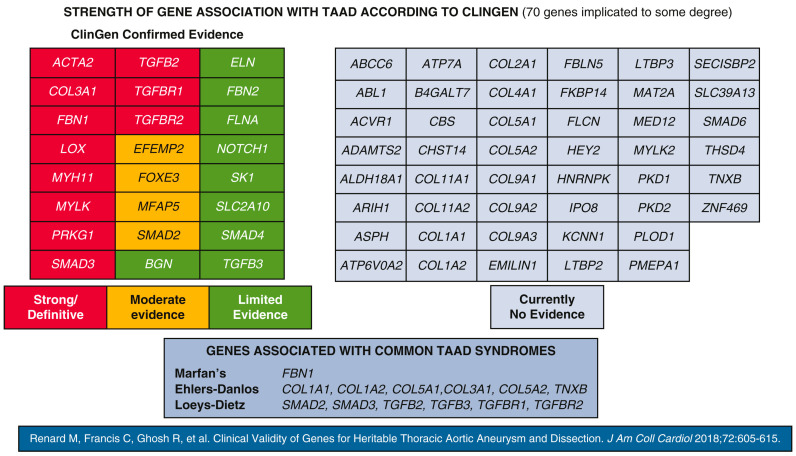
Seventy genes associated with thoracic aortic aneurysm and dissection, grouped by the strength of association according to the Clinical Genome Resource (ClinGen) framework from 2018. Reproduced from Elefteriades et al. [18] with permission from Elsevier.

The ClinGen Aortopathy Working Group recognises 70 genes associated with TAAs, 11 of which are pathogenic or likely pathogenic variants [75]. These genes encode proteins integral to the elastin–contractile unit, including those involved in SMC contraction, TGF-β signalling, ECM adhesion, and SMC metabolism (Figure 3). Mutations in these genes are prevalent in patients and families exhibiting systemic features of syndromic HTADs (e.g., Marfan syndrome, Loeys–Dietz syndrome, or Ehlers–Danlos syndrome). In approx. 20% of syndromic and non-syndromic HTAD cases, a genetic cause was identified, whereas in around 5% of sporadic TAA and AAA cases, pathogenic defects in aneurysm-related genes were responsible.

While both genetic susceptibilities and environmental factors are important in the development of aneurysms, their interplay appears to play an even larger role and remains incompletely understood. This observation emerged from studies of individual- and family-level defects in aneurysm-related genes. Indeed, defects in aneurysm-related genes put individuals and families at higher risk of TAAs, AAAs, or both, but they cannot explain the localisation, timing, or severity of the disease. 

### 3.10. Pathogenic Variants in Genes Encoding ECM Components

The FBN1 gene, associated with Marfan syndrome, was the first HTAD gene identified. FBN1 encodes fibrillin-1, a large cysteine-rich glycoprotein that forms microfibrils around elastin fibres. Structurally, fibrillin-1 features epidermal growth factor (EGF)-like motifs interspersed with TGF-β1 binding protein motifs. Cysteines in the 47 EGF-like motifs form disulfide bonds, whereas those in the 7 TGF-β1 motifs stabilise fibrillin-1’s quaternary structure. Approximately 2000 FBN1 pathogenic variants have been identified in MFS patients, disrupting the structural attachment of elastin fibres to SMCs and reducing fibrillin-1 content in microfibrils [13]. Most pathogenic variants change a single base pair, affecting the protein’s shape and function, which reduces fibrillin-1 production or makes it more vulnerable to degradation [76]. FBN1 pathogenic variants can induce SMC phenotype switching, enhance MMP activity, and diminish TGF-β sequestration, promoting non-canonical TGF-β signalling [77,78]. However, variants in the FBN1 gene were identified in healthy individuals, suggesting that its presence does not necessarily translate to Marfan syndrome development [79].

The stability of elastin and collagen depends on lysine oxidation, mediated by the copper-dependent amino oxidase lysyl oxidase (LOX). Deficiency or loss of LOX activity leads to aneurysms in the aortic root and ascending aorta, as well as skeletal features resembling those of MFS [80], and AAAs in mice [81]. Pathogenic variants in pro-collagen genes, such as COL3A1, are implicated in vascular Ehlers–Danlos syndrome. COL3A1 encodes type III pro-collagen, secreted and cleaved to form ECM [82]. Other COL genes, such as COL5A1, encoding collagen V, or COL1A2, encoding collagen 1, have been linked to TAAs in patients with Ehlers–Danlos syndrome [83,84].

### 3.11. Pathogenic Variants in TGF-β Signalling Pathway Genes

Alterations in TGF-β signalling, including mutations in receptors (TGFBR1, TGFBR2), ligands (TGFB2), and downstream signalling factors (SMADs), have been identified in TAA and dissection cases [13,54]. Mutations in the TGFBR1, TGFBR2, TGFB2, and SMAD3 genes cause Loeys–Dietz syndrome, characterised by systemic extravascular features [67,85]. Clinical features of this syndrome vary among individuals of the same family, suggesting a complex interplay between genetic susceptibility and other risk factors that are not fully understood yet. These pathogenic variants in aneurysm-related genes impair canonical TGF-β signalling, disrupt SMC differentiation, and reduce the expression of contractile proteins. Alterations in the TGF-β pathway occur in both genetically derived and sporadic aneurysms. In TGFBR2-mutant patients, fibroblasts fail to differentiate into mature myofibroblasts in response to TGF-β stimulation, suggesting compromised myofibroblast activation [86]. Evidence from mice studies suggests that TGF-β signalling protects against aneurysm formation by promoting aortic wall homeostasis. Mice with deficiencies in Smad3, Smad4, and TGFB2 develop aneurysms, and TGFBR2 deletion causes TAAD in postnatal mice [24,49], while Baas et al. [87] found that variants in TGFBR1 and TGFBR2 were associated with increased AAA risk. 

The propotease FURIN is a novel gene that predisposes to TAA and AAA, and is of particular interest since its identification in a cohort of unrelated patients with AAAs [88]. FURIN cleaves several proprotein substrates, e.g., profibrillin, pro TGF-β, and procollagens. It can impair proTGF-β processing and TGF-β maturation, compromising TGF-β signalling, and has also been linked to hypertension and cardiovascular disease [89,90]. 

### 3.12. Pathogenic Defects in Genes Coding for the SMC Contractile Unit

The SMC contractile apparatus consists of α-actin-thin and myosin-thick filaments. ACTA2 encodes α-actin, constituting approximately 40% of SMC protein content, and polymerises into thin filaments. Pathogenic variants disrupt amino acids in actin subunits, compromising the structural integrity necessary to propagate pulse pressures, resulting in impaired SMC contraction. Approximately 14% of patients with non-syndromic HTADs harbour ACTA2 defects, presenting heightened risk for coronary artery disease and ischemic strokes [91,92].

Thick filaments comprise myosin heavy chain dimers and associated regulatory (RLC) and essential light chains. SMC contraction and relaxation depend on RLC phosphorylation. Pathogenic variants of the MYH11-gene impair myosin heavy chain polymerisation and filament formation, diminishing force generation. Patients with MYH11 defects exhibit TAAs, dissections, and patent ductus arteriosus. 

Phosphorylation can be altered by defects in MYLK and PRKG1, which are essential for contraction. MYLK encodes myosin light chain kinase, promoting RLC phosphorylation and SMC contraction. MYLK defects predominantly cause aortic dissections without preceding aneurysmal enlargement [93], while PRKG1 encodes type 1 cGMP-dependent protein kinase (PKG-1), mediating SMC relaxation through dephosphorylation. Gain-of-function pathogenic variants of PRKG1 impair SMC contractility or induce excessive relaxation, commonly resulting in HTADs with descending thoracic aorta dissection in young patients (63% at a mean age of 31 years) [94].

## 4. Clinical Implications

**Figure 4:**
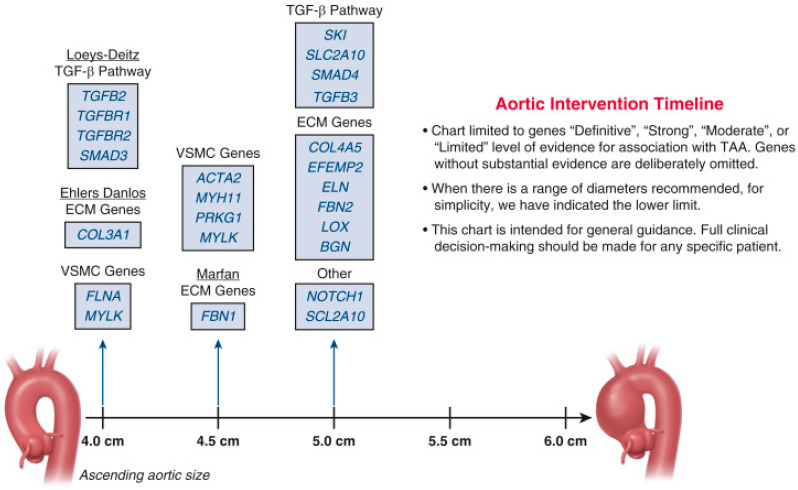
Timeline for surgical intervention in ascending TAAs. This chart outlines the genetic mutations known to cause TAAs, with arrows indicating the general aortic diameter threshold at which surgical intervention is recommended for each mutation. The chart is intended to support, but not replace, comprehensive clinical decision-making. TAA: thoracic aortic aneurysm. Reproduced from Elefteriades et al. [18] with permission from Elsevier.

**Figure 5:**
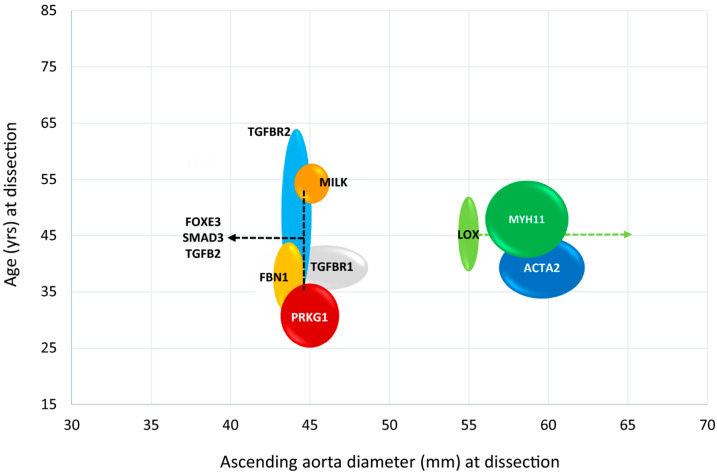
Schematic representation of genetic mutations with associated age and ascending aortic diameter at dissection. The width of the circles and lines represents the SD in age and diameter. Data were obtained from studies included in the systematic review. No numerical data were available for patients with aortic dissection associated with NOTCH1 or MFAP5 mutations, and patients with MAT2A mutations did not experience aortic dissections. SD: standard deviation. Reproduced from Mariscalo et al. [102].

**Figure 6:**
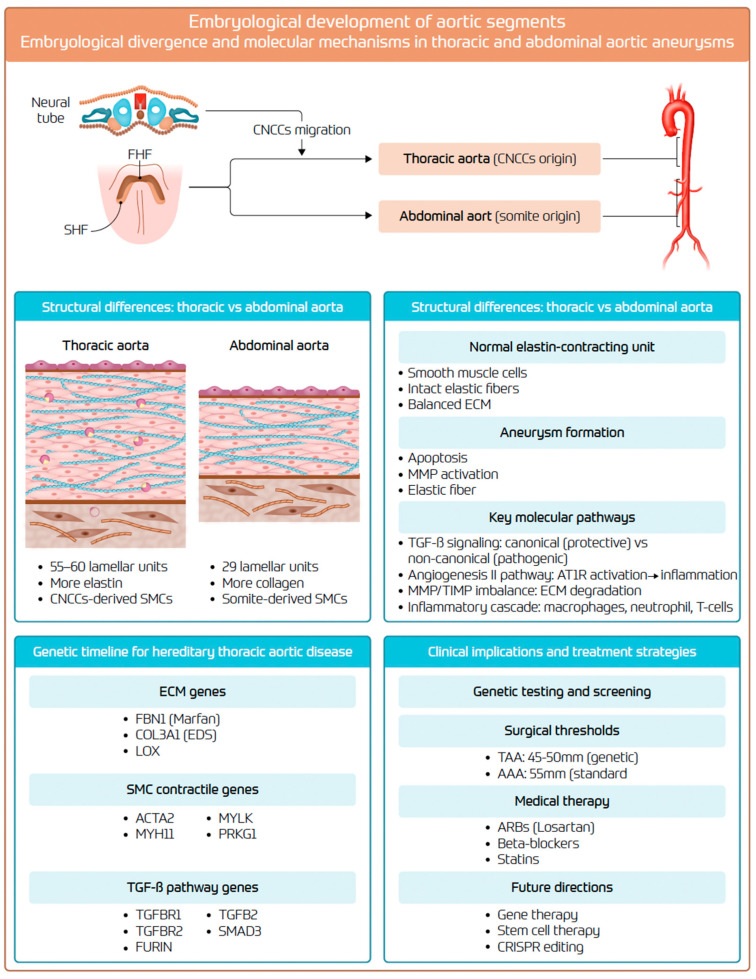
Integrative overview of the “aortic organ” linking embryologic lineage, regional wall architecture, key molecular pathways, genetic determinants, and clinical management in TAAs and AAAs. AAA: abdominal aortic aneurysm; TAA: thoracic aortic aneurysm; CNCCs: cardiac neural crest cells; SHF: second heart field; FHF: first heart field; SMC: smooth muscle cell; ECM: extracellular matrix; MMP: matrix metalloproteinase; TIMP: tissue inhibitor of metalloproteinases; TGF-β: transforming growth factor-β; AT1R: angiotensin II type-1 receptor; ARB: angiotensin receptor blocker; EDS: Ehlers–Danlos syndrome; CRISPR: clustered regularly interspaced short palindromic repeats.

Extensive research into embryological, histological, and functional aortic heterogeneity have significantly advanced our understanding of the molecular mechanisms and genetic determinants underlying both sporadic and hereditary aneurysms. The increased accessibility and reduced costs of whole-exome sequencing have facilitated broader genetic diagnosis, influencing clinical guidelines for surgical intervention by incorporating genetic factors alongside aneurysm size, patient age, and comorbidities [8,95]. The “genetic timeline for surgery”, as proposed by Elefteriades et al. (Figure 4) [18], outlines surgical intervention thresholds based on genetic penetrance and disease severity. Preventive surgical repair should be considered based on disease severity, individual patient characteristics, and institutional expertise (Figure 5) [8]. Current pharmacological interventions supporting aneurysm management include AT-II receptor blockers [96], beta-blockers [97], and intensive lipid-lowering treatment [30]. Additionally, mesenchymal stem cells have shown potential therapeutic benefit in preclinical testing by reducing aneurysm progression through anti-inflammatory effects and preservation of extracellular matrix integrity [98,99,100]. An integrative schematic is provided in Figure 6. Finally, although thoracic and abdominal aneurysms arise in different segments, they share core histopathologic hallmarks and show substantial overlap in genetic and familial susceptibility. Clinically, this supports the careful documentation of family history, consideration of whole-aorta imaging in selected patients, and targeted cascade screening of first-degree relatives when appropriate, since it has been shown that screening reduces aorta-related mortality [16,101].

## 5. Knowledge Gaps and Future Directions

Despite substantial progress in developmental biology, molecular genetics, and translational research, several key knowledge gaps persist, hindering the complete clinical translation of emerging insights into the pathophysiology of aortic aneurysms. Bridging these gaps will require sustained interdisciplinary collaboration among basic scientists, clinicians, geneticists, computational biologists, and patient communities to translate molecular understanding into meaningful clinical outcomes. First, a significant challenge lies in the clinical interpretation of rare and novel genetic variants. While the current ACMG/ClinGen classifications, including pathogenic, likely pathogenic, benign, and variants of uncertain significance, offer a foundational framework, many aortopathy-associated variants remain difficult to categorise with confidence [103,104]. Reliance on population allele frequencies, evolutionary conservation, and in silico prediction tools has limited precision. Furthermore, functional validation, which is often dependent on time-intensive cellular assays or multigenerational clinical correlation, presents logistical challenges. Still, recent advances in genome editing technologies, particularly CRISPR-based knock-in/knock-out models, combined with single-cell multi-omic platforms, offer a promising route toward real-time variant adjudication and clinical applicability [105,106]. Second, unlike TAAs, which often exhibit high-penetrance monogenic mutations, AAAs are primarily driven by complex interactions between polygenic susceptibility and environmental exposures such as hypertension and smoking. To address this, large-scale genome-wide association studies and diverse, multi-ancestry biobank initiatives are essential for refining polygenic risk scores and for capturing gene–environment interactions that could inform individualised surveillance and prevention strategies. Third, a further unmet need is the development of spatially resolved molecular atlases of the “aortic organ.” Currently, there is a lack of segment-specific transcriptomic and proteomic profiling across different developmental stages and disease states. Mapping the molecular signatures of the ascending, arch, descending, and abdominal aorta in both healthy and aneurysmal conditions could reveal lineage-specific vulnerabilities and guide segment-tailored therapeutic interventions. Fourth, the reliance on maximal diameter as the primary determinant of surgical indication is increasingly recognised as an imperfect surrogate for rupture or dissection risk. Future strategies should incorporate circulating biomarkers, such as elastin degradation products and inflammatory mediators, along with advanced imaging modalities like PET-MRI and computational modelling of wall stress, to detect early signs of biomechanical instability independent of aneurysm size. Fifth, in terms of pharmacologic therapy, there remains a paucity of effective disease-modifying agents for non-syndromic aortopathies. While ARBs have demonstrated benefits in Marfan syndrome, there is an urgent need to explore other targets. This includes small-molecule inhibitors of non-canonical TGF-β signalling, safer MMP inhibitors, and RNA-based therapies such as antisense oligonucleotides, all of which merit evaluation in genetically and phenotypically stratified patient populations. Finally, the integration of artificial intelligence and data science into clinical practice is poised to revolutionise the field. Machine learning algorithms trained on multidimensional datasets, including clinical characteristics, genetic profiles, imaging data, and haemodynamic parameters, have the potential to construct individualised risk trajectories and optimise the timing of surgical intervention. However, robust validation through international registries and pragmatic clinical trials remains a key priority.

## 6. Conclusions

Aortic aneurysms are complex vascular diseases with potentially life-threatening complications arising from the intersection of developmental biology, regional biomechanics, and molecular signalling. Over the past two decades, significant advances in understanding have been achieved, ranging from morphological classification to molecular and genetic understanding, clarifying why thoracic and abdominal segments differ in their susceptibility to disease. Increasingly detailed genetic profiling of thoracic aortopathy has enabled genotype-guided surgical thresholds and risk stratification, while polygenic models are beginning to inform abdominal disease surveillance. As the limitations of diameter-based criteria become more evident, interest is growing with regard to biomarker-driven assessment, advanced imaging, and computational modelling to anticipate dissection and rupture more accurately. In parallel, novel RNA-based and pharmacologic therapies are being explored for disease modification. Future progress will depend on integrating these biological, technological, and clinical advances into unified care pathways. Translational research, large-scale registries, and interdisciplinary collaboration will be essential to delivering individualised, mechanism-informed care and ultimately improving outcomes for patients with aortic aneurysms.

## Data Availability

Not applicable.

## References

[1] Huang X., Wang Z., Shen Z., Lei F., Liu Y.M., Chen Z., Qin J.J., Liu H., Ji Y.X., Zhang P. (2022). Projection of global burden and risk factors for aortic aneurysm—Timely warning for greater emphasis on managing blood pressure. Ann. Med..

[2] Wang Z., You Y., Yin Z., Bao Q., Lei S., Yu J., Xie C., Ye F., Xie X. (2022). Burden of Aortic Aneurysm and Its Attributable Risk Factors from 1990 to 2019: An Analysis of the Global Burden of Disease Study 2019. Front. Cardiovasc. Med..

[3] Oladokun D., Patterson B.O., Sobocinski J., Karthikesalingam A., Loftus I., Thompson M.M., Holt P.J. (2016). Systematic Review of the Growth Rates and Influencing Factors in Thoracic Aortic Aneurysms. Eur. J. Vasc. Endovasc. Surg..

[4] Lindholt J.S., Sogaard R. (2017). Population screening and intervention for vascular disease in Danish men (VIVA): A randomised controlled trial. Lancet.

[5] Wanhainen A., Hultgren R., Linne A., Holst J., Gottsater A., Langenskiold M., Smidfelt K., Björck M., Svensjö S., Swedish Aneurysm Screening Study Group (SASS) (2016). Outcome of the Swedish Nationwide Abdominal Aortic Aneurysm Screening Program. Circulation.

[6] Oliver-Williams C., Sweeting M.J., Turton G., Parkin D., Cooper D., Rodd C., Thompson S.G., Earnshaw J.J. (2018). Lessons learned about prevalence and growth rates of abdominal aortic aneurysms from a 25-year ultrasound population screening programme. Br. J. Surg..

[7] Marcaccio C.L., Schermerhorn M.L. (2021). Epidemiology of abdominal aortic aneurysms. Semin. Vasc. Surg..

[8] Czerny M., Grabenwoger M., Berger T., Aboyans V., Della Corte A., Chen E.P., Desai N.D., Dumfarth J., Elefteriades J.A., Etz C.D. (2024). EACTS/STS Guidelines for diagnosing and treating acute and chronic syndromes of the aortic organ. Eur. J. Cardio-Thorac. Surg..

[9] Obel L.M., Diederichsen A.C., Steffensen F.H., Frost L., Lambrechtsen J., Busk M., Urbonaviciene G., Egstrup K., Karon M., Rasmussen L.M. (2021). Population-Based Risk Factors for Ascending, Arch, Descending, and Abdominal Aortic Dilations for 60–74-Year-Old Individuals. J. Am. Coll. Cardiol..

[10] Bonser R.S., Ranasinghe A.M., Loubani M., Evans J.D., Thalji N.M., Bachet J.E., Carrel T.P., Czerny M., Di Bartolomeo R., Grabenwöger M. (2011). Evidence, lack of evidence, controversy, and debate in the provision and performance of the surgery of acute type A aortic dissection. J. Am. Coll. Cardiol..

[11] Pacini D., Di Marco L., Fortuna D., Belotti L.M., Gabbieri D., Zussa C., Pigini F., Contini A., Barattoni M.C., De Palma R. (2013). Acute aortic dissection: Epidemiology and outcomes. Int. J. Cardiol..

[12] Yamaguchi T., Nakai M., Yano T., Matsuyama M., Yoshino H., Miyamoto Y., Sumita Y., Matsuda H., Inoue Y., Okita Y. (2021). Population-based incidence and outcomes of acute aortic dissection in Japan. Eur. Heart J. Acute Cardiovasc. Care.

[13] Pinard A., Jones G.T., Milewicz D.M. (2019). Genetics of Thoracic and Abdominal Aortic Diseases. Circ. Res..

[14] Wu J., Zafar M.A., Liu Y., Chen J.F., Li Y., Ziganshin B.A., Ellauzi H., Mukherjee S.K., Rizzo J.A., Elefteriades J.A. (2023). Fate of the unoperated ascending thoracic aortic aneurysm: Three-decade experience from the Aortic Institute at Yale University. Eur. Heart J..

[15] Harik L., Leith J., Rahouma M., Cancelli G., Rossi C.S., Soletti G.J., Balaram S.K., Lau C., Girardi L., Gaudino M. (2025). Association between social vulnerability and clinical outcomes after proximal aortic surgery. Eur. J. Cardio-Thorac. Surg..

[16] Mazzolai L., Teixido-Tura G., Lanzi S., Boc V., Bossone E., Brodmann M., Bura-Riviere A., De Backer J., Deglise S., Della Corte A. (2024). 2024 ESC Guidelines for the management of peripheral arterial and aortic diseases. Eur. Heart J..

[17] Isselbacher E.M. (2005). Thoracic and abdominal aortic aneurysms. Circulation.

[18] Elefteriades J.A., Zafar M.A., Ziganshin B.A. (2024). Genetics of aortic aneurysm disease: 10 key points for the practitioner. JTCVS Open.

[19] Liyew W.A., Adane F., Wondemagegn A.T., Tsehay B., Deml Y.A., Abdu H.M., Animaw Z. (2024). Roles of cardiac neural crest cells in cardiovascular development and associated congenital defects-an integrated review. Transl. Res. Anat..

[20] Waldo K.L., Hutson M.R., Ward C.C., Zdanowicz M., Stadt H.A., Kumiski D., Abu-Issa R., Kirby M.L. (2005). Secondary heart field contributes myocardium and smooth muscle to the arterial pole of the developing heart. Dev. Biol..

[21] Majesky M.W. (2007). Developmental basis of vascular smooth muscle diversity. Arter. Thromb. Vasc. Biol..

[22] Kirby M.L., Waldo K.L. (1995). Neural crest and cardiovascular patterning. Circ. Res..

[23] Majesky M.W., Dong X.R., Hoglund V., Mahoney W.M., Daum G. (2011). The adventitia: A dynamic interface containing resident progenitor cells. Arter. Thromb. Vasc. Biol..

[24] Shen Y.H., LeMaire S.A. (2017). Molecular pathogenesis of genetic and sporadic aortic aneurysms and dissections. Curr. Probl. Surg..

[25] Wolinsky H., Glagov S. (1967). A lamellar unit of aortic medial structure and function in mammals. Circ. Res..

[26] Wagenseil J.E., Mecham R.P. (2012). Elastin in large artery stiffness and hypertension. J. Cardiovasc. Transl. Res..

[27] Wolinsky H., Glagov S. (1969). Comparison of abdominal and thoracic aortic medial structure in mammals. Deviation of man from the usual pattern. Circ. Res..

[28] Ruddy J.M., Jones J.A., Spinale F.G., Ikonomidis J.S. (2008). Regional heterogeneity within the aorta: Relevance to aneurysm disease. J. Thorac. Cardiovasc. Surg..

[29] Wolinsky H. (1970). Comparison of medial growth of human thoracic and abdominal aortas. Circ. Res..

[30] Ejiri J., Inoue N., Tsukube T., Munezane T., Hino Y., Kobayashi S., Hirati K.-I., Kawashima S., Imajoh-Ohmi S., Hayashi Y. (2003). Oxidative stress in the pathogenesis of thoracic aortic aneurysm: Protective role of statin and angiotensin II type 1 receptor blocker. Cardiovasc. Res..

[31] Jia L.X., Zhang W.M., Zhang H.J., Li T.T., Wang Y.L., Qin Y.W., Gu H., Du J. (2015). Mechanical stretch-induced endoplasmic reticulum stress, apoptosis and inflammation contribute to thoracic aortic aneurysm and dissection. J. Pathol..

[32] Domagala D., Data K., Szyller H., Farzaneh M., Mozdziak P., Wozniak S., Zabel M., Dziegiel P., Kempisty B. (2024). Cellular, Molecular and Clinical Aspects of Aortic Aneurysm-Vascular Physiology and Pathophysiology. Cells.

[33] Rateri D.L., Moorleghen J.J., Balakrishnan A., Owens A.P., Howatt D.A., Subramanian V., Poduri A., Charnigo R., Cassis L.A., Daugherty A. (2011). Endothelial cell-specific deficiency of Ang II type 1a receptors attenuates Ang II-induced ascending aortic aneurysms in LDL receptor^-/-^ mice. Circ. Res..

[34] Fan L.M., Douglas G., Bendall J.K., McNeill E., Crabtree M.J., Hale A.B., Mai A., Li J.M., McAteer M.A., Schneider J.E. (2014). Endothelial cell-specific reactive oxygen species production increases susceptibility to aortic dissection. Circulation.

[35] DeRoo E., Stranz A., Yang H., Hsieh M., Se C., Zhou T. (2022). Endothelial Dysfunction in the Pathogenesis of Abdominal Aortic Aneurysm. Biomolecules.

[36] Malashicheva A., Kostina D., Kostina A., Irtyuga O., Voronkina I., Smagina L., Ignatieva E., Gavriluk N., Uspensky V., Moiseeva O. (2016). Phenotypic and Functional Changes of Endothelial and Smooth Muscle Cells in Thoracic Aortic Aneurysms. Int. J. Vasc. Med..

[37] Yang X., Xu C., Yao F., Ding Q., Liu H., Luo C., Wang D., Huang J., Li Z., Schen Y. (2023). Targeting endothelial tight junctions to predict and protect thoracic aortic aneurysm and dissection. Eur. Heart J..

[38] Rong J.X., Shapiro M., Trogan E., Fisher E.A. (2003). Transdifferentiation of mouse aortic smooth muscle cells to a macrophage-like state after cholesterol loading. Proc. Natl. Acad. Sci. USA.

[39] Lin C.J., Keating C., Roth R., Caliskan Y., Nazzal M., Exil V., DiPaolo R., Verma D.R., Harjai K., Zayed M. (2024). Distinct Patterns of Smooth Muscle Phenotypic Modulation in Thoracic and Abdominal Aortic Aneurysms. J. Cardiovasc. Dev. Dis..

[40] Hu Y., Cai Z., He B. (2023). Smooth Muscle Heterogeneity and Plasticity in Health and Aortic Aneurysmal Disease. Int. J. Mol. Sci..

[41] Chen P.Y., Qin L., Li G., Malagon-Lopez J., Wang Z., Bergaya S., Gujja S., Caulk A.W., Murtada S.I., Zhang X. (2020). Smooth Muscle Cell Reprogramming in Aortic Aneurysms. Cell Stem Cell.

[42] Mellak S., Ait-Oufella H., Esposito B., Loyer X., Poirier M., Tedder T.F., Tedgui A., Mallat Z., Potteaux S. (2015). Angiotensin II mobilizes spleen monocytes to promote the development of abdominal aortic aneurysm in *Apoe*^−/−^ mice. Arter. Thromb. Vasc. Biol..

[43] Furubayashi K., Takai S., Jin D., Miyazaki M., Katsumata T., Inagaki S., Kimura M., Tanaka K., Nishimoto M., Fukumoto H. (2008). Chymase activates promatrix metalloproteinase-9 in human abdominal aortic aneurysm. Clin. Chim. Acta.

[44] Elkhal A., Rodriguez Cetina Biefer H., Heinbokel T., Uehara H., Quante M., Seyda M., Schuitenmaker J.M., Krenzien F., Camacho V., de la Fuente M.A. (2016). NAD(+) regulates Treg cell fate and promotes allograft survival via a systemic IL-10 production that is CD4(+) CD25(+) Foxp3(+) T cells independent. Sci. Rep..

[45] Galis Z.S., Khatri J.J. (2002). Matrix Metalloproteinases in Vascular Remodeling and Atherogenesis. Circ. Res..

[46] Rabkin S.W. (2014). Differential expression of MMP-2, MMP-9 and TIMP proteins in thoracic aortic aneurysm—Comparison with and without bicuspid aortic valve: A meta-analysis. Vasa.

[47] Goodall S., Crowther M., Hemingway D.M., Bell P.R., Thompson M.M. (2001). Ubiquitous elevation of matrix metalloproteinase-2 expression in the vasculature of patients with abdominal aneurysms. Circulation.

[48] Martin-Alonso M., Garcia-Redondo A.B., Guo D., Camafeita E., Martinez F., Alfranca A., Mendez-Berbero N., Pollan A., Sanchez-Camacho C., Denhardt D.T. (2015). Deficiency of MMP17/MT4-MMP proteolytic activity predisposes to aortic aneurysm in mice. Circ. Res..

[49] Ito S., Lu H.S., Daugherty A., Sawada H. (2022). Embryonic Heterogeneity of Smooth Muscle Cells in the Complex Mechanisms of Thoracic Aortic Aneurysms. Genes.

[50] Wang L., Song J., Yang Z., Zhang H., Wang Y., Liu J., Wang S., Shi J., Tong X. (2025). SERCA2 dysfunction accelerates angiotensin II-induced aortic aneurysm and atherosclerosis by induction of oxidative stress in aortic smooth muscle cells. J. Mol. Cell. Cardiol..

[51] Das A.A., Waldeck-Weiermair M., Yadav S., Spyropoulos F., Pandey A., Dutta T., Convington T.A., Michel T. (2025). Differential aortic aneurysm formation provoked by chemogenetic oxidative stress. J. Clin. Investig..

[52] Ballinger S.W. (2005). Mitochondrial dysfunction in cardiovascular disease. Free Radic. Biol. Med..

[53] Habashi J.P., Judge D.P., Holm T.M., Cohn R.D., Loeys B.L., Cooper T.K., Myers L., Klein E.C., Liu G., Calvi C. (2006). Losartan, an AT1 antagonist, prevents aortic aneurysm in a mouse model of Marfan syndrome. Science.

[54] El-Hamamsy I., Yacoub M.H. (2009). Cellular and molecular mechanisms of thoracic aortic aneurysms. Nat. Rev. Cardiol..

[55] Dai J., Losy F., Guinault A.M., Pages C., Anegon I., Desgranges P., Becquemin J.P., Allaire E. (2005). Overexpression of transforming growth factor-beta1 stabilizes already-formed aortic aneurysms: A first approach to induction of functional healing by endovascular gene therapy. Circulation.

[56] Holm T.M., Habashi J.P., Doyle J.J., Bedja D., Chen Y., van Erp C., Lindsay M.E., Kim D., Schoenhoff F., Cohn R.D. (2011). Noncanonical TGFbeta signaling contributes to aortic aneurysm progression in Marfan syndrome mice. Science.

[57] Iske J., El Fatimy R., Nian Y., Ghouzlani A., Eskandari S.K., Cetina Biefer H.R., Vasudevan A., Elkhal A. (2024). NAD(+) prevents septic shock-induced death by non-canonical inflammasome blockade and IL-10 cytokine production in macrophages. Elife.

[58] Stone J.R., Bruneval P., Angelini A., Bartoloni G., Basso C., Batoroeva L., Buja L.M., Butany J., d’Amati G., Fallon J.T. (2015). Consensus statement on surgical pathology of the aorta from the Society for Cardiovascular Pathology and the Association for European Cardiovascular Pathology: I. Inflammatory diseases. Cardiovasc. Pathol..

[59] Alcorn H.G., Wolfson S.K., Sutton-Tyrrell K., Kuller L.H., O’Leary D. (1996). Risk factors for abdominal aortic aneurysms in older adults enrolled in The Cardiovascular Health Study. Arter. Thromb. Vasc. Biol..

[60] Forsdahl S.H., Singh K., Solberg S., Jacobsen B.K. (2009). Risk factors for abdominal aortic aneurysms: A 7-year prospective study: The Tromso Study, 1994–2001. Circulation.

[61] Naydeck B.L., Sutton-Tyrrell K., Schiller K.D., Newman A.B., Kuller L.H. (1999). Prevalence and risk factors for abdominal aortic aneurysms in older adults with and without isolated systolic hypertension. Am. J. Cardiol..

[62] Johnsen S.H., Forsdahl S.H., Singh K., Jacobsen B.K. (2010). Atherosclerosis in abdominal aortic aneurysms: A causal event or a process running in parallel? The Tromso study. Arter. Thromb. Vasc. Biol..

[63] Liu J., Lu H., Howatt D.A., Balakrishnan A., Moorleghen J.J., Sorci-Thomas M., Cassis L.A., Daugherty A. (2015). Associations of ApoAI and ApoB-containing lipoproteins with AngII-induced abdominal aortic aneurysms in mice. Arter. Thromb. Vasc. Biol..

[64] Maegdefessel L., Spin J.M., Tsao P.S. (2014). New ways to dismantle a ticking time bomb: MicroRNA 712/205 and abdominal aortic aneurysm development. Arter. Thromb. Vasc. Biol..

[65] Merk D.R., Chin J.T., Dake B.A., Maegdefessel L., Miller M.O., Kimura N., Tsao P.S., Iosef C., Berry G.J., Mohr F.W. (2012). miR-29b participates in early aneurysm development in Marfan syndrome. Circ. Res..

[66] Winski G., Chernogubova E., Busch A., Eken S.M., Jin H., Lindquist Liljeqvist M., Khan T., Backlund A., Poloschi V., Roy J. (2025). MicroRNA-15a-5p mediates abdominal aortic aneurysm progression and serves as a potential diagnostic and prognostic circulating biomarker. Commun Med..

[67] Cho M.J., Lee M.R., Park J.G. (2023). Aortic aneurysms: Current pathogenesis and therapeutic targets. Exp. Mol. Med..

[68] Bararu Bojan Bararu I., Plesoianu C.E., Badulescu O.V., Vladeanu M.C., Badescu M.C., Iliescu D., Bojan A., Ciocoiu M. (2023). Molecular and Cellular Mechanisms Involved in Aortic Wall Aneurysm Development. Diagnostics.

[69] Ziganshin B.A., Bailey A.E., Coons C., Dykas D., Charilaou P., Tanriverdi L.H., Lie L., Tranquilli M., Bale A.E., Elefteriades J.A. (2015). Routine Genetic Testing for Thoracic Aortic Aneurysm and Dissection in a Clinical Setting. Ann. Thorac. Surg..

[70] Wahlgren C.M., Larsson E., Magnusson P.K., Hultgren R., Swedenborg J. (2010). Genetic and environmental contributions to abdominal aortic aneurysm development in a twin population. J. Vasc. Surg..

[71] Liu H., de Bruin J.L., Verhagen H.J.M., Roos-Hesselink J.W., Bekkers J.A., Brüggenwirth H.T., van Beusekom H.M.M., Majoor-Krakauer D.F., ASIJ (2025). Whole aorta imaging shows increased risk for thoracic aortic aneurysms and dilatations in relatives of abdominal aortic aneurysm patients. J. Vasc. Surg..

[72] Roychowdhury T., Klarin D., Levin M.G., Spin J.M., Rhee Y.H., Deng A., Headley C.A., Tsao N.L., Gellatly C., Zuber V. (2023). Genome-wide association meta-analysis identifies risk loci for abdominal aortic aneurysm and highlights PCSK9 as a therapeutic target. Nat. Genet..

[73] Zheng S., Tsao P.S., Pan C. (2024). Abdominal aortic aneurysm and cardiometabolic traits share strong genetic susceptibility to lipid metabolism and inflammation. Nat. Commun..

[74] Golledge J., Lu H.S., Shah S. (2024). Protein convertase subtilisin/kexin type 9 as a drug target for abdominal aortic aneurysm. Curr. Opin. Lipidol..

[75] Renard M., Francis C., Ghosh R., Scott A.F., Witmer P.D., Ades L.C., Andelfinger G.U., Arnaud P., Boileau C., Callewaert B.L. (2018). Clinical Validity of Genes for Heritable Thoracic Aortic Aneurysm and Dissection. J. Am. Coll. Cardiol..

[76] Pinard A., Salgado D., Desvignes J.P., Rai G., Hanna N., Arnaud P., Guien C., Martinez M., Faivre L., Jondeau G. (2016). WES/WGS Reporting of Mutations from Cardiovascular “Actionable” Genes in Clinical Practice: A Key Role for UMD Knowledgebases in the Era of Big Databases. Hum. Mutat..

[77] Chung A.W., Au Yeung K., Sandor G.G., Judge D.P., Dietz H.C., van Breemen C. (2007). Loss of elastic fiber integrity and reduction of vascular smooth muscle contraction resulting from the upregulated activities of matrix metalloproteinase-2 and -9 in the thoracic aortic aneurysm in Marfan syndrome. Circ. Res..

[78] Neptune E.R., Frischmeyer P.A., Arking D.E., Myers L., Bunton T.E., Gayraud B., Ramirez F., Sakai L.Y., Dietz H.C. (2003). Dysregulation of TGF-beta activation contributes to pathogenesis in Marfan syndrome. Nat. Genet..

[79] Walker S., Bunyan D.J., Thomas H.B., Kesim Y., Kershaw C.J., Holloway J., Wai H., Day M., Smith C.L., Hawkes G. (2025). Utility of genome sequencing and group-enrichment to support splice variant interpretation in Marfan syndrome. Genet. Med..

[80] Guo D.C., Regalado E.S., Gong L., Duan X., Santos-Cortez R.L., Arnaud P., Ren Z., Cai B., Hostetler E.M., Moran R. (2016). LOX Mutations Predispose to Thoracic Aortic Aneurysms and Dissections. Circ. Res..

[81] Gyftopoulos A., Ziganshin B.A., Elefteriades J.A., Ochoa Chaar C.I. (2023). Comparison of Genes Associated with Thoracic and Abdominal Aortic Aneurysms. AORTA.

[82] Superti-Furga A., Gugler E., Gitzelmann R., Steinmann B. (1988). Ehlers-Danlos syndrome type IV: A multi-exon deletion in one of the two COL3A1 alleles affecting structure, stability, and processing of type III procollagen. J. Biol. Chem..

[83] Monroe G.R., Harakalova M., van der Crabben S.N., Majoor-Krakauer D., Bertoli-Avella A.M., Moll F.L., Oranen B.I., Dooijes D., Vink A., Knoers N.V. (2015). Familial Ehlers-Danlos syndrome with lethal arterial events caused by a mutation in *COL5A1*. Am. J. Med. Genet. A.

[84] Schwarze U., Hata R., McKusick V.A., Shinkai H., Hoyme H.E., Pyeritz R.E., Byers P.H. (2004). Rare autosomal recessive cardiac valvular form of Ehlers-Danlos syndrome results from mutations in the *COL1A2* gene that activate the nonsense-mediated RNA decay pathway. Am. J. Hum. Genet..

[85] Loeys B.L., Chen J., Neptune E.R., Judge D.P., Podowski M., Holm T., Mayers J., Leitch C.C., Katsanis N., Sharifi N. (2005). A syndrome of altered cardiovascular, craniofacial, neurocognitive and skeletal development caused by mutations in *TGFBR1* or *TGFBR2*. Nat. Genet..

[86] Inamoto S., Kwartler C.S., Lafont A.L., Liang Y.Y., Fadulu V.T., Duraisamy S., Willing M., Estrera A., Safi H., Hannibal M.V. (2010). *TGFBR2* mutations alter smooth muscle cell phenotype and predispose to thoracic aortic aneurysms and dissections. Cardiovasc. Res..

[87] Baas A.F., Medic J., van’t Slot R., de Vries J.P., van Sambeek M.R., Geelkerken B.H., Boll B.P., Grobbee D.E., Wijmenga C., Ruigrok Y.M. (2010). Association study of single nucleotide polymorphisms on chromosome 19q13 with abdominal aortic aneurysm. Angiology.

[88] He Z., IJpma A.S., Vreeken D., Heijsman D., Rosier K., Verhagen H.J.M., de Bruin J.L., Bruggenwirth H.T., Roos-Hesselink J.W., Bekkers J.A. (2024). The proprotein convertase FURIN is a novel aneurysm predisposition gene impairing TGF-beta signalling. Cardiovasc. Res..

[89] Plaimauer B., Mohr G., Wernhart W., Himmelspach M., Dorner F., Schlokat U. (2001). ‘Shed’ furin: Mapping of the cleavage determinants and identification of its C-terminus. Biochem. J..

[90] Ehret G.B., Munroe P.B., Rice K.M., Bochud M., Johnson A.D., Chasman D.I., Smith A.V., Tobin M.D., Verwoert C., International Consortium for Blood Pressure Genome-Wide Association Studies (2011). Genetic variants in novel pathways influence blood pressure and cardiovascular disease risk. Nature.

[91] Guo D.C., Pannu H., Tran-Fadulu V., Papke C.L., Yu R.K., Avidan N., Bourgeois S., Estrera A.L., Safi H.J., Sparks E. (2007). Mutations in smooth muscle alpha-actin (*ACTA2*) lead to thoracic aortic aneurysms and dissections. Nat. Genet..

[92] Milewicz D.M., Ostergaard J.R., Ala-Kokko L.M., Khan N., Grange D.K., Mendoza-Londono R., Bradley T.J., Haskins Olney A., Ades L., Maher J.F. (2010). De novo *ACTA2* mutation causes a novel syndrome of multisystemic smooth muscle dysfunction. Am. J. Med. Genet. A.

[93] Shalata A., Mahroom M., Milewicz D.M., Limin G., Kassum F., Badarna K., Tarabeih N., Assy N., Fell R., Cohen H. (2018). Fatal thoracic aortic aneurysm and dissection in a large family with a novel MYLK gene mutation: Delineation of the clinical phenotype. Orphanet J. Rare Dis..

[94] Guo D.C., Regalado E., Casteel D.E., Santos-Cortez R.L., Gong L., Kim J.J., Dyack S., Horne A.G., Chang G., Jondeau G. (2013). Recurrent gain-of-function mutation in *PRKG1* causes thoracic aortic aneurysms and acute aortic dissections. Am. J. Hum. Genet..

[95] Stark Z., Dolman L., Manolio T.A., Ozenberger B., Hill S.L., Caulfied M.J., Levy Y., Glazer D., Wilson J., Lawler M. (2019). Integrating Genomics into Healthcare: A Global Responsibility. Am. J. Hum. Genet..

[96] Kuang S.Q., Geng L., Prakash S.K., Cao J.M., Guo S., Villamizar C., Kwartler C.S., Peters A.M., Brasier A.R., Milewich D.M. (2013). Aortic remodeling after transverse aortic constriction in mice is attenuated with AT_1_ receptor blockade. Arter. Thromb. Vasc. Biol..

[97] Pitcher A., Spata E., Emberson J., Davies K., Halls H., Holland L., Wilson K., Reith C., Child A.H., Clayton T. (2022). Angiotensin receptor blockers and beta blockers in Marfan syndrome: An individual patient data meta-analysis of randomised trials. Lancet.

[98] Turnbull I.C., Hadri L., Rapti K., Sadek M., Liang L., Shin H.J., Costa K.D., Marin M.M., Hajjar R.J., Faries P.L. (2011). Aortic implantation of mesenchymal stem cells after aneurysm injury in a porcine model. J. Surg. Res..

[99] Hashizume R., Yamawaki-Ogata A., Ueda Y., Wagner W.R., Narita Y. (2011). Mesenchymal stem cells attenuate angiotensin II-induced aortic aneurysm growth in apolipoprotein E-deficient mice. J. Vasc. Surg..

[100] Schneider F., Saucy F., de Blic R., Dai J., Mohand F., Rouard H., Ricco J.B., Becquemin J.P., Gervais M., Allaire E. (2013). Bone marrow mesenchymal stem cells stabilize already-formed aortic aneurysms more efficiently than vascular smooth muscle cells in a rat model. Eur. J. Vasc. Endovasc. Surg..

[101] Verhagen J.M.A., Kempers M., Cozijnsen L., Bouma B.J., Duijnhouwer A.L., Post J.G., Hilhorst-Hofstee Y., Bekkers S.C.A.M., Kerstjens-Frederikse W.S., van Brakel T.J. (2018). Expert consensus recommendations on the cardiogenetic care for patients with thoracic aortic disease and their first-degree relatives. Int. J. Cardiol..

[102] Mariscalco G., Debiec R., Elefteriades J.A., Samani N.J., Murphy G.J. (2018). Systematic Review of Studies That Have Evaluated Screening Tests in Relatives of Patients Affected by Nonsyndromic Thoracic Aortic Disease. J. Am. Heart Assoc..

[103] Richards S., Aziz N., Bale S., Bick D., Das S., Gastier-Foster J., Grody W.W., Hegde M., Lyon E., Spector E. (2015). Standards and guidelines for the interpretation of sequence variants: A joint consensus recommendation of the American College of Medical Genetics and Genomics and the Association for Molecular Pathology. Genet. Med..

[104] Masson E., Zou W.B., Genin E., Cooper D.N., Le Gac G., Fichou Y., Pu N., Rebours V., Ferec C., Liao Z. (2022). Expanding ACMG variant classification guidelines into a general framework. Hum. Genom..

[105] Raghavan A., Pirruccello J.P., Ellinor P.T., Lindsay M.E. (2024). Using Genomics to Identify Novel Therapeutic Targets for Aortic Disease. Arter. Thromb. Vasc. Biol..

[106] Liu N., Olson E.N. (2022). CRISPR Modeling and Correction of Cardiovascular Disease. Circ. Res..

